# Tumor-Derived Exosomal Non-Coding RNAs: The Emerging Mechanisms and Potential Clinical Applications in Breast Cancer

**DOI:** 10.3389/fonc.2021.738945

**Published:** 2021-10-11

**Authors:** Yi Yi, Min Wu, Hong Zeng, Weijie Hu, Chongru Zhao, Mingchen Xiong, Wenchang Lv, Pei Deng, Qi Zhang, Yiping Wu

**Affiliations:** Department of Plastic Surgery, Tongji Hospital, Tongji Medical College, Huazhong University of Science and Technology, Wuhan, China

**Keywords:** breast cancer, exosomes, non-coding RNAs, biomarkers, drug resistance

## Abstract

Breast cancer (BC) is the most frequent malignancy and is ranking the leading cause of cancer-related death among women worldwide. At present, BC is still an intricate challenge confronted with high invasion, metastasis, drug resistance, and recurrence rate. Exosomes are membrane-enclosed extracellular vesicles with the lipid bilayer and recently have been confirmed as significant mediators of tumor cells to communicate with surrounding cells in the tumor microenvironment. As very important orchestrators, non-coding RNAs (ncRNAs) are aberrantly expressed and participate in regulating gene expression in multiple human cancers, while the most reported ncRNAs within exosomes in BC are microRNAs (miRNAs), long-noncoding RNAs (lncRNAs), and circular RNAs (circRNAs). Notably, ncRNAs containing exosomes are novel frontiers to shape malignant behaviors in recipient BC cells such as angiogenesis, immunoregulation, proliferation, and migration. It means that tumor-derived ncRNAs-containing exosomes are pluripotent carriers with intriguing and elaborate roles in BC progression *via* complex mechanisms. The ncRNAs in exosomes are usually excavated based on specific de-regulated expression verified by RNA sequencing, bioinformatic analyses, and PCR experiments. Here, this article will elucidate the recent existing research on the functions and mechanisms of tumor-derived exosomal miRNA, lncRNA, circRNA in BC, especially in BC cell proliferation, metastasis, immunoregulation, and drug resistance. Moreover, these tumor-derived exosomal ncRNAs that existed in blood samples are proved to be excellent diagnostic biomarkers for improving diagnosis and prognosis. The in-depth understanding of tumor-derived exosomal ncRNAs in BC will provide further insights for elucidating the BC oncogenesis and progress and exploring novel therapeutic strategies for combating BC.

## Introduction

Breast cancer (BC) is one of the most common malignant tumors among females. As a highly heterogeneous cancer, BC can be divided into various distinct subtypes according to gene expression profiling, such as luminal A, luminal B, human epidermal growth factor receptor 2 (HER2)-positive and basal-like (triple-negative) BC (1). Despite there are tremendous advances in early detection, diagnosis, and therapy strategies, BC is still one of the primary reasons for cancer-related deaths in females due to the poor prognosis caused by tumor metastasis and recurrence (2). Over the past decades, numerous studies have attempted to illuminate the underlying mechanisms leading to BC oncogenesis, proliferation, and metastasis (3). However, the accurate mechanisms remain elusive by now. The establishment of identifying the key molecules and mechanisms is a prerequisite for the development of predictive and diagnostic biomarkers and innovative treatments for overcoming BC.

**Figure 1:**
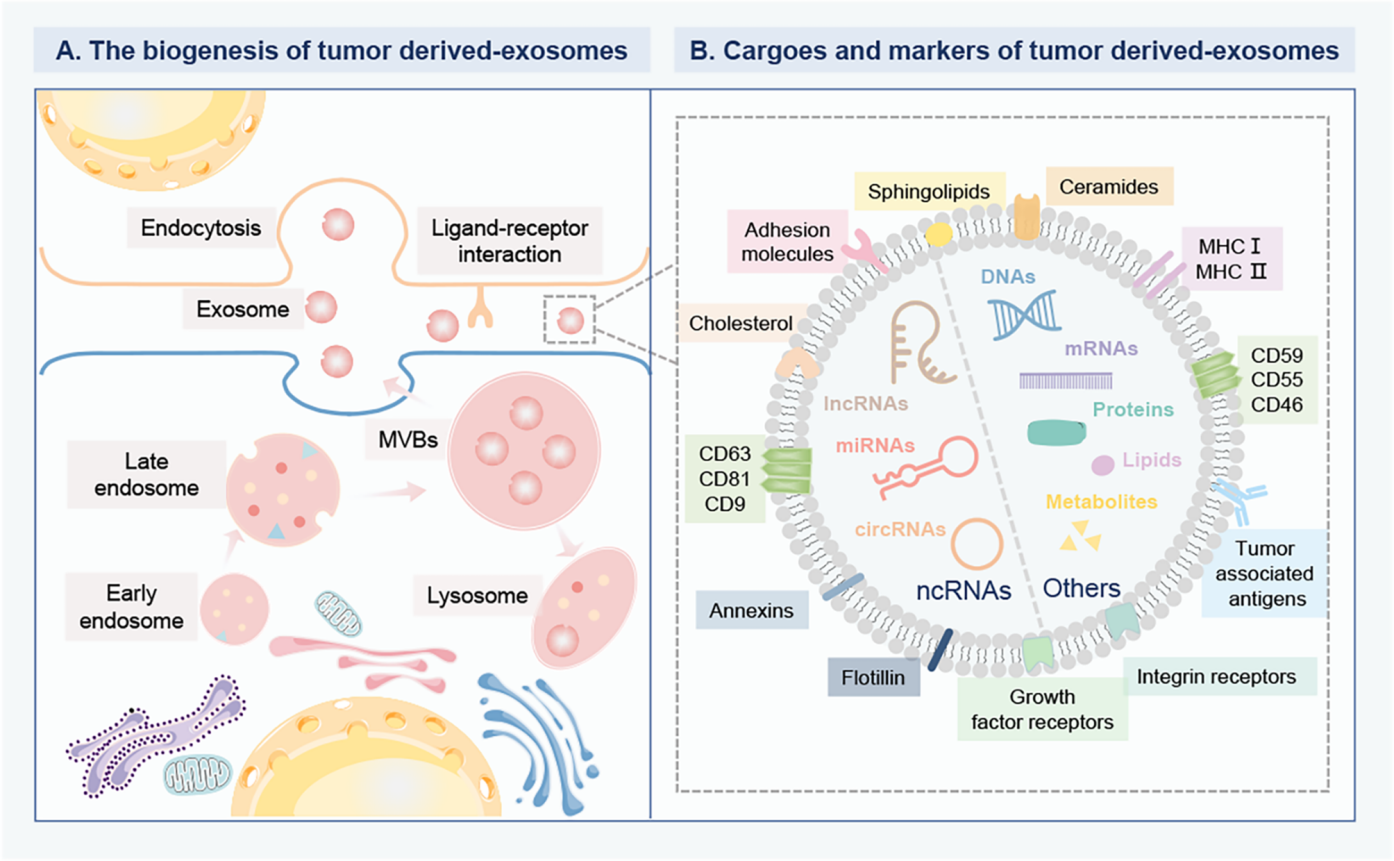
The biogenesis and composition of tumor derived-exosomes in BC. **(A)** The biogenesis of tumor derived-exosomes. The early endosomes are formed by the inward germination of the plasma membrane, and evolve into the late endosomes and then into MVBs. Some MVBs are degraded by fusion with lysosomes, while the others are trafficked to fuse with the plasma membrane to release exosomes into the extracellular environment. Then, the recipient cells can internalize exosomes for gene modification through different mechanisms, including direct internalization membrane fusion, large pinocytosis, phagocytosis and endocytosis. Exosomes can deliver multiple bioactive cargoes from parent cells to recipient cells. **(B)** The cargo profile and biomarkers of tumor derived-exosomes. The specific cargoes are encapsulated within exosomes, mainly including various proteins, DNAs, metabolites, lipids, mRNAs and ncRNAs, such as miRNAs, lncRNA, circRNAs. The specific membrane proteins anchor on the surface of exosomes as biomarkers, including a conserved set of proteins (caveolins, clathrin, transferrin receptors), tetraspanins (CD63, CD81, CD9), MHC I and II, tumor-associated antigens, growth factor receptors, and integrin receptors. These biomarkers are involved in the interaction between ligands and receptors on target cells. Breast cancer, BC; Multivesicular bodies, MVBs; Messenger RNAs, mRNAs; Non-coding RNAs, ncRNAs; MicroRNA, miRNAs; Long-noncoding RNA, lncRNA; circular RNAs, circRNAs; Major histocompatibility complex class-I and II, MHC I and II.

Exosomes are specifically defined as membrane-enclosed extracellular vesicles (EVs) with particle diameter sizes of 40 nm to 160 nm, which are released from cells upon fusion of the multivesicular body with the plasma membrane (4). Exosomes are widely distributed in the body fluids, such as blood, urine, saliva, amniotic fluid, cerebrospinal fluid, lymph, and bile, under both healthy and pathological conditions (5). The common exosomal biomarkers are associated with the exosome biogenesis, release, and fusion events, including a conserved set of proteins (caveolins, clathrin, transferrin receptors), tetraspanins (CD63, CD81, CD9) (6). Exosomes can deliver cargos into the extracellular space mainly containing soluble proteins (enzymes, cytokines, chemokines) and membrane-bound proteins, DNAs, messenger RNAs (mRNAs), non-coding RNAs (ncRNAs), lipids, and chemical messengers, which can reflect their cell of origin (7). Understanding the carried molecular and cellular properties of exosome cargos is important to offer benefits for identifying the key molecular events in disease initiation, occurrence, and progression. Exosome secretion is one of the emerging significant mechanisms for tumor cells to reciprocal communicate with surrounding cells. Recent studies have shown that tumor-released exosomes could shuttle and interplay between seeds and soil to induce tumor cell malignancy and organotypic metastasis (8). It is worth mentioning that the exosomes can transfer a variety of photogenic factors to the target organ to induce inflammation foci formation and thus lead to the recruitment of bone marrow precursors and the remodeling of the pre-metastatic niches (9). The metastasizing process is closely related to the surface-specific integrin on tumor exosomes. Therefore, the tumor exosomes are the important link in coordinating the complex multi-stage process of tumor metastasis. Due to their stability and disease-specific cargos, tumor-derived exosomes in blood circulation and in blood cell-surface-associated origination emerging as valuable targets for monitoring tumor progression (10). For example, Laktionov et al. analyzed the plasma-separated exosome from healthy females and BC patients by ultracentrifugation and found that the plasmic exosomal miR-103, miR-191, miR-195 that was associated with the fraction of red blood cells were more precisely to discriminate the BC in comparison to the cell-free exosomes circulating in plasma, confirming that better diagnostic value of isolated miRNA from cell-derived exosomes (11). Gonzalez et al. confirmed that the concentration of exosomes in BC patients with stages I, III, and IV was significantly higher compared with healthy donors (12). Determining the molecular properties of these cargos in exosomes remains a fundamental milestone toward understanding the molecular heterogeneity in BC as well as improving the application in theranostic strategies in BC (**Figure 1**).

A large number of ncRNAs and their functionalities have generated lots of interest in BC. ncRNAs make up the vast majority of the eukaryotic transcriptome. Although with no or only limited protein-coding ability, ncRNAs can be transcribed into various RNA species to exert vital functions mainly responsible for post-transcriptional regulation (13). Dysregulation of ncRNAs has been proven to orchestrate BC progress by inducing proliferation, invasion, metastasis, cachexia of tumor cells (14). Importantly, a wide range of ncRNAs including microRNA (miRNAs), long-noncoding RNA (lncRNA), circular RNAs (circRNAs), piwi-interacting RNAs (piRNAs), small nucleolar RNA, can be synergistically packaged into tumor-derived exosomes (15). The most abundant ncRNAs within exosomes are miRNA, lncRNA, and circRNA. miRNAs, belonging to a class of small ncRNAs ranging from 19-24 nt in length, can regulate gene expression posttranscriptionally *via* either translation repression or mRNA degradation by binding to their complementary sites in the 3’-untranslated region (UTR) of mRNAs (16). lncRNAs are broadly defined as a class of ncRNAs transcripts for gene regulations with a length longer than 200 nucleotides (17). CircRNAs, are a new type of functional ncRNAs characterized by a covalent single-strand loop, accompanied by several notable characteristics, including high abundance, species diversity, structural stability, evolutionary conservation, localization, and specificity (18). Tumors are regulated by different genetic and epigenetic events of endogenous and exogenous sources. As a new way of cellular communication, exosomes are capable of carrying arrays of these oncogenic proteins and ncRNAs and then are internalized by the neighbor cells. As a consequence, the RNA-containing exosomes can then relay cellular signals from the donor cells to regulate the activities of the recipient cells. Especially, the exosomal ncRNAs are crucial players in the establishment of the metastatic niche by communication between cancer cells and normal cells (19).

As discussed above, tumor-derived exosomal ncRNAs are essential to modify the cancer cell phenotypes locally or systemically through the exosome-dependant exchange of genetic information. Thus, this article has deciphered the recent existing research on the roles and mechanisms of tumor-derived exosomal miRNA, lncRNA, circRNA in BC, especially in aspects of BC cell proliferation, metastasis, and immunoregulation. It also emphasizes the clinical implication of tumor-derived exosomal miRNA, lncRNA, circRNA in BC diagnosis, and drug resistance. Overall, an in-depth understanding of tumor-derived exosomal ncRNAs in BC will provide profound insight into the BC oncogenesis and progress and screen novel strategies for therapeutic interventions of BC.

## Exosomal miRNAs

Importantly, the miRNA carried by exosomes was shown to transfer to recipient cells through direct uptake, playing a gene silencing effect to fine-tune target expression like endogenic miRNAs. Moreover, exosomal miRNAs can even impact the environment surrounding the tumor, influence the extracellular matrix (ECM) as well as immune system activation and recruitment.

### Exosomal miRNAs in BC Growth

Stevic et al. determined the miRNA profiles in circulating exosomes of BC patients using quantitative miRNA array, emphasizing that several miRNAs were differently expressed in exosomes of either HER2-positive or triple-negative breast cancer (TNBC) patients compared with healthy women, such as miR-27a/b, miR-335, miR-365, miR-376c, miR-382, miR-422a, miR-433, and miR-628 (20). It clarified a network of deregulated exosomal miRNAs with specific expression patterns in HER2-positive and TNBC patients that were also associated with clinicopathological parameters and pathological complete response within each BC subtype (20). The packaging miRNA sets in BC exosomes could be served as potential diagnostic markers for monitoring BC. By using the same methods, Ni et al. also identified the exosomal miRNAs in 111 BC patients, 42 ductal carcinomas *in situ* (DCIS) patients and 39 healthy women (21). The results confirmed the different signatures of abnormal miR-16, miR-30b, and miR-93 in exosomes from BC and DCIS patients were associated with the particular biology of breast tumors. DCIS has the potential to develop invasive ductal carcinoma (IDC). Yoshikawa et al. posed that exosomal miR−223−3p levels separated from IDC patient plasma were higher than healthy control and DCIS (22). miR-223-3p promoted the invasion of BC cells, and exosomal miR-223-3p might be a minimally invasive biomarker for the selection of patients with invasion from DSIC patients.

By analyzing the miRNA profiles, Chen et al. found the participation of exosomal miR−130a and miR−425 in MCF−7/S cell viability, which was associated with malignant cell proliferation pathways, such as TOR, ErbB, MAPK and TGF-β (23). Besides, MCF-7 cells transfected miR-223-3p significantly promoted BC cell proliferation and cell invasion ability (22). By comprehensive analysis of microarray datasets from the public Gene Expression Omnibus (GEO) database, Xin et al. screened that exosomal miR-455-5p and miR-1255a resulted in poor prognosis and proved to be novel therapeutic targets for BC (24). miR-455-5p and miR-1255a could be transported from the BC cells to non-malignant recipient cells *via* inhibiting the expression of CDKN1B and SMAD4. Xia et al. indicated that the anticancer functions of halofuginone contributed to the shuttled exosomal miR-31 that could modulate the growth of the MCF-7 cells by specifically targeting the histone deacetylase 2 (HDAC2), which increased the levels of cyclin-dependent kinases 2 (CDK2) and cyclin D1 and suppressed the expression of p21 (25). Midori et al. showed that exosomes isolated from the HCC1806 TNBC cells were able to induce proliferation and drug resistance on the non-tumorigenic MCF10A breast cells, possibly mediated by the changes in 138 genes and 70 miRNAs expression, affecting including PI3K/AKT, MAPK, and HIF1A (26). Among the up-regulated differentially expressed (DE) miRNAs, miR-155-5p could regulate targets like APC, HSD17B12, MYC, SMAD1 and SMAD3, and act as an oncogenic miRNA involved in BC initiation and progress.

More and more researches have revealed that in the tumor microenvironment (TME), the normal fibroblasts (NFs) can transform into cancer-associated fibroblasts (CAFs), which could be recruited and activated by paracrine factors released from BC cells. A variety of juxtacrine and paracrine interactions represented by exosomal contents, occur between BC cells and CAFs to direct tumor progression. Importantly, Baroni et al. reported that in couples of primary NFs/CAFs isolated from patients with four BC subtypes, only miR-9 exhibited a significantly higher level just in triple-negative CAFs compared with the normal counterpart. In addition, tumor-secreted miR-9 *via* exosomes was transferred to NFs and increased cell motility, and NFs transfected with miR-9 significantly promoted the *in vivo* tumor growth (27). It was proved that miR-3613-3p was up-regulated in exosomes from fibroblasts educated by TGF-β1 and the fibroblasts of BC tissues (28). The cellular functions showed that the downregulation of miR-3613-3p in CAFs exosomes suppressed BC cell proliferation, reactive oxygen species (ROS) production, and metastasis by targeting SOCS2 (28). The study demonstrated that activated CAF exosomes played an oncogenic role in BC *via* miR-3613-3p. A very interesting study by Yan et al. indicated a mechanistic model involving BC EVs-encapsulated miR-105, which was induced by the oncoprotein MYC in BC cells and, in turn, activated MYC signaling in CAFs to induce a metabolic program (29). When nutrition was adequate, the CAF reprogrammed by miR-105 enhanced glucose and glutamine metabolism, thereby fueling nearby cancer cells. Hence, the capacity of miR-105-mediated metabolic reprogramming of CAFs contributed to sustained tumor growth by regulating the shared metabolic environment (29). Shah et al. reported that CAFs conditioned media from different BC subtypes contained diverse miRNA profiles and CAF-secreted secreted miR-221/222 involved in exosomes mediated estrogen receptor (ER) repression (30). The results strongly indicated that CAFs performed hierarchical paracrine interactions with BC cells through hMAPK-miRNA to drive the ER-negative BC phenotype. Therefore, the dynamic interplay between CAFs and BC cells could be mediated by exosomes to transfer oncogenic miRNAs cargos to remodel cancer progression.

### Exosomal miRNAs in BC Metastasis

**Figure 2:**
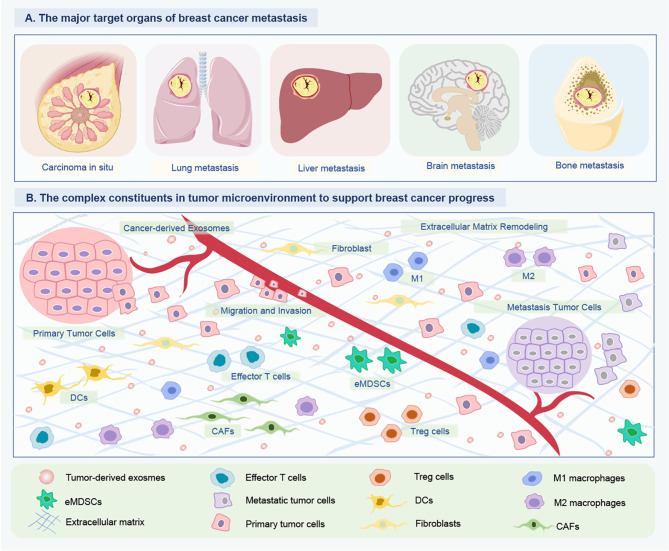
Tumor-derived exosomal ncRNAs mediate intercellular communication between BC cells and their surrounding microenvironment. **(A)** The major target organs of BC metastasis. The organotropic metastasis of BC is mainly from breast to the lung, liver, bone and brain. **(B)** The complex constituents in tumor microenvironment of BC. To support tumor growth, the BC cells of tumor *in situ* can modify their surrounding microenvironment and distant environment *via* releasing multiple cytokine and ncRNA-containing exosomes. The crosstalk between BC and host organ promotes the formation of pre-metastasis niches, and is enhanced partially by exosomes released by BC cells. Consequently, tumor-derived exosome could promote BC cells metastasizing to distant organs. Besides, these exosomal ncRNAs can educate immune cells (DCs, eMDSCs, Tregs, M1/M2 macrophages) and remodel the extracellular matrix (CAFs) to create a favorable condition for BC growth and metastasis, and even drug resistance. ncRNAs, Non-coding RNAs; BC, Breast cancer; DCs, Dendritic cells; eMDSCs, Early-stage myeloid-derived suppressor cells; CAFs, Cancer-associated fibroblasts.

Metastasis is a multi-step process, in which the cancer cells acquire alterations contributing to surpass their programmed behavior to disseminate from the primary tumor, penetrate the blood circulation, and eventually spread into distant tissues (**Figure 2A**). By microarray analysis, Kruger et al. identified several oncogenic miRNAs with higher amounts in tumor exosomes, including miR-130a/miR-328 in MDA-MB-231 cells, and miR-106b/miR-34a in MCF-7, which might link to the enhanced metastatic property of BC (31). Wei et al. demonstrated that miR-128 in BC tumor-derived exosomes was able to negatively regulate the Bax protein in MCF-7 recipient cells and inhibit cell proliferation (32). miR-1246 was up-regulated in BC patients, especially in those with metastatic BC cell lines. Li et al. confirmed that the transferred miR-1246 could promote invasion in normal HMLE cells partially targeting CCNG2 by binding to its 3’-UTR (33). In the plasma sample of BC, exosomal miR-222 was highly expressed in advanced BC patients with lymphatic metastasis, as well as closely correlated with the high aggressivity of BC cell lines (34). Mechanistically, exosomal miR-222 intercellular transferring promoted migration and invasion of the recipient BC cells by repressing PDLIM2 expression and consequently enhancing NF-κB (34). *In vitro* assay, Kia et al. showed that miR-9 and miR-155 containing exosomes in highly metastatic MDA-MB-231 cells induced metastatic phenotype in recipient cells in non-metastatic MCF-7, with decreased PTEN and DUSP14 expression. This study introduced a novel route by preventing exosome secretion from metastatic and cancerous cells to prevent BC metastasis (35). The upregulation of RAB22A was associated with BC progression and lymph node metastasis. Sun et al. identified a signature of RAB22A and miR-193b that exhibited a negative association in metastatic as opposed to the surrounding normal cells. Besides, the oncogenic RAB22A, regulated by miR-193b, affected the exosome-mediated growth and invasion of the recipient BC cells (36). Singh et al. verified that miR-10b was highly expressed in metastatic BC MDA-MB-231 cells as compared to non-metastatic BC cells or non-malignant breast cells. Oncogenic miR-10b, secreted by BC cells, could influence the adjacent and distant normal cells, leading to beneficial results for tumor development and progression (37). In the process of metastatic spread, BC acquires the ability to transmigrate through blood vessels by inducing changes in the endothelial barrier. As reported by Modica et al., the exosomal miR-939 in TNBC cells increased tumor cell trans-endothelial migration and regulated cadherin 5 (CDH5) in endothelial cells. This work demonstrated that tumor-derived exosomal miR-939 participated in the extracellular pro-tumorigenic function and was associated with worse prognosis in TNBCs (38).

The lymphatic vessels (LV) within the TME are leaky compared to blood vessels and are considered a primary route of cancer dissemination. Kim et al. reported that ELK3 expressed in lymphatic endothelial cells (LECs) contributed to the dissemination of cancer cells during tumor growth by providing exosomal oncogenic miRNAs to tumor cells (39). Furthermore, conditioned medium from ELK3-suppressed LECs lost the ability to promote the migration and invasion of BC cells *in vitro*, by targeting miR-503-3p, miR-4269, and miR-30e-3p (39). Hypoxic tumors may communicate with surrounding tumor and non-tumor cells to induce more malignant phenotypes *via* exosomes. In both *in vitro* and *in vivo* visualization, Jung et al. found that exosomes could target localize in tumors, suggesting tumor tropism of exosomes (40). It also verified that hypoxic BC cell exosomes could transfer miRNA-210 to normoxic tumor/or endothelial cells and that miR-210 was associated with the expression of vascular remodeling related genes Ephrin A3 and PTP1B, to promote angiogenesis in recipient cells (40). These results indicated that cellular miRNAs components from hypoxic cancer cells spread to adjacent cancer cells in the TME *via* exosomes.

BC cells preferentially metastasize to specific organs, known as “organotropic metastasis”, which is influenced by BC subtypes. The crosstalk between BC and host organ promotes the formation of pre-metastasis niches and is enhanced by factors (such as exosomes) released by cancer cells before they reach the host organ. Kong et al. found that the lower levels of exosome-derived miR-130a-3p were related to lymph node metastasis and advanced tumor-node-metastasis (TNM) stage in BC (41). Overexpression of miR-130a-3p in breast cancer stem cells (BCSCs) inhibited cell proliferation, migration, and invasion potentially *via* downregulation of RAB5B, and the silencing of miR-130a-3p was of the opposite effects. miR-130a-3p might act as a disease progression monitoring biomarker and therapeutic target in BC. Rodríguez et al. showed that exosomes released by T47D-CXCR4 were enriched in specific mRNAs related to stemness and metastasis, supporting the view that BC cells with stem-like properties possessed the concomitant metastatic behavior, and their exosomes could stimulate tumor progression and metastasis (42). MDA-MB-231-derived exosomes releasing miR-454 disrupted the Wnt pathway by targeting PRRT2, thereby promoting the biological properties of cancer stem cells (CSCs) *in vitro* and ovarian cancer cell growth *in vivo* (43).

TNBC tends to transfer to the lungs and brain, which is different from other types that usually transfer to bones and soft tissues. The ITGβ4 positive-tumor exosomes could be used as natural targeting nanocarriers for protective miRNA-126 delivery, which was capable of recognizing A549 cells in the blood and availably escaping from the immune surveillance system *in vitro* (44). When tested in a lung metastasis model, miRNA-231 exosomes resulted in an efficacious impact on inhibiting the formulation of lung metastasis *in vivo*. In the *in vitro* assay, Paryan et al. found that miR-9 and miR-155 were among the over-expressed miRNAs in highly metastatic TNBC cells and their exosomes, by targeting PTEN and DUSP14 tumor suppressor genes respectively, which was confirmed by bioinformatic and luciferase assay (45). BC bone metastasis is difficult to cure, accompanied by bone pain, bone fractures, hypercalcemia, and spinal cord compression. miR-20a-5p was highly expressed in BC tumor tissues and the exosomes of MDA-MB-231 cells. MDA-MB-231 cell-derived exosomes transferred miR-20a-5p to the primary murine bone marrow macrophages and facilitated the osteoclastogenesis *via* targeting SRCIN, providing evidence for the development of exosome or miR-20a-5p targeted therapeutic intervention in BC bone metastasis (46). Sharma et al. developed a special therapy for brain metastasis which delivered athermal radiofrequency electromagnetic fields that were amplitude-modulated at BC-specific frequencies (BCF). Brain-tropic variant cells showed higher levels of exosomal miR-1246, while BCF reduced the expression of miR-1246 in exosomes secreted by brain metastatic cells to suppress angiogenesis in the brain microenvironment. It strongly suggested that miR-1246 might orchestrate the angiogenic niche in the brain and potentially serve as a biomarker of brain metastasis (47).

## Exosomal lncRNAs

lncRNAs are a category of cellular RNAs with various secondary structures to specifically bind to proteins and nucleic acids, based on the principle of complementary base pairing (48). Exosomal lncRNAs commonly function as competing RNA sponges or endogenous RNAs (ceRNAs) to regulate gene expression. Aberrant expression of tumor-derived lncRNA served as a momentous part in BC cell proliferation, invasion, and metastasis.

### Exosomal lncRNAs in BC Growth

lncRNAs can be delivered into extracellular space and adjust the function of neighboring or distant cells in a paracrine manner. Shao et al. found that lncRNA CASC9 was significantly up-regulated in both BC tissues and cell lines, thus playing an oncogenic role in BC (49). lncRNA CASC9 positively regulated checkpoint kinase 1 (CHK1) by competitively binding to the miR-195/497 cluster, thereby accelerated BC cell proliferation, accelerated cell cycle progression, and inhibited cell apoptosis (49). It has also been reported that the circulatory exosomal HOTAIR plays a role in the bladder, glioblastoma, and cervical cancer. Similarly, Zhang et al. confirmed that circulatory exosomal HOTAIR was present in cell lines as well as blood samples of recruited BC patients (50). The expression of exosomal HOTAIR was positively correlated with the status of the receptor tyrosine kinase (RTK) ErbB2 in tumor tissues. lncRNA MALAT1 was also up-regulated in BC tissues compared and the serum levels of cell-free MALAT1 could significantly differentiate between BC patients and volunteers (51). Besides, MALAT1 was significantly highly expressed in BC cell exosomes, while exosome-mediated delivery of MALAT1 induced cell proliferation in BC (51). The exosomes secreted from CAFs reprogram the metabolic pathways after the exosome internalization by the tumor cells. Li et al. provided evidence that CAF-exosomal lncRNA SNHG3 was abnormally increased in BC patients. Mechanistically, SNHG3 functioned as a miR-330-5p sponge to positively regulate PKM expression, inhibited mitochondrial oxidative phosphorylation, increased glycolysis carboxylation, and enhanced BC cell proliferation (52). Thus, SNHG3 could play an important role in the development and progression of BC. Collectively, these studies support the oncogenic potential of exosomal lncRNA communication between cancer cells and TME.

### Exosomal lncRNAs in BC Metastasis

Cancer metastasis relies on the interactions between cancer cells and different components of TME. Exosomal lncRNAs have been implicated as novel mediators of intercellular communication within the primary TME and metastasizing niche. Lu et al. established a brain metastasis BC cell model by injecting MDA-MB-231-Luc-D3H2LN cells into immunodeficient female mice, posing that lncRNAGS1-600G8.5 was highly expressed in exosomes derived from readily metastasized to the brain, compared to the lncRNA in exosomes from low metastatic cells (53). Exosomal lncRNA GS1-600G8.5 disrupted the blood-brain barrier (BBB) and promoted the BC passage across the BBB, potentially by targeting tight junction proteins. Moreover, Xing et al. profiled lncRNAs in brain metastatic tumors from patients with BC and found that the lncRNA XIST was significantly downregulated in these tissues (54). Downregulation of lncRNA XIST activated three distinct pathways, epithelial-mesenchymal transition (EMT), MSN/c-Met, and release of exosomal miR-503, thus knockout of XIST in mice mammary glands promoted the primary tumor growth as well as metastasis in the brain. Importantly, they also found that fludarabine was a potential therapeutic agent for the treatment of patients with breast cancer brain metastasis (BCBM) that had low levels of XIST expression (54). Feng et al. also observed that BC exosomes could boost tumor pulmonary metastasis *via* mediating dysregulation of lncRNAs (55). There were 64 co-increased lncRNAs and 8 co-decreased lncRNAs in lung fibroblasts treated with BC exosomes (55). The results demonstrated that dysregulation of BC-secreted exosomal lncRNAs could accelerate the lung metastasis of the tumor by regulating the malignant transformation of lung fibroblasts, leading to pre-metastatic niche formation.

## Exosomal circRNAs

circRNAs are highly conserved and relatively stable compared with other linear counterparts (56). Based on the composition, circRNAs can be generally classified into 4 types: exonic circRNAs, exon-intron circRNAs, circular intronic RNAs, and tRNA intronic circRNAs (57). Most circRNAs can function as a miRNA sponge or RNA binding protein, and can also regulate alternative splicing and modulate the expression of their parental genes. It has been reported that circRNAs are highly abundant and stable in exosomes, particularly in tumor-derived exosomes. Notably, circRNAs are capable of longer half-life resulted in more frequent incorporation into exosomes than linear RNAs (57). It is expectable that crucial regulatory functions of exosomal circRNAs in various behaviors of cancer, such as tumorigenesis, differentiation, proliferation, and metastasis.

By RNA sequence profiling, Yang et al. found that 646 circRNAs were significantly upregulated and 157 circRNAs were significantly downregulated in serum-exosomes of BC patients. It also identified the higher expression of hsa-circRNA-0088088 and hsa-circRNA-00000751 and lower expression of hsa-circRNA-00005795 by reverse transcription-polymerase chain reaction (RT-PCR) (58). Predicted potential circRNA-miRNA interaction networks showed that hsa-circRNA-0088088 and hsa-circRNA-0005795 might function as ceRNAs and were associated with several cancer-related pathways, such as the Wnt, estrogen, TGF-β, and FoxO signaling pathways (58). Amorim et al. presented a next-generation sequencing (NGS)-based transcriptome view of the miRNA and circRNA landscape of two breast-derived cell lines, HB4a and C5.2, in the context of HER2 upregulation, demonstrating that identification of hundreds of circRNAs in these cells as well as their exosome-containing EVs (59). Wang identified 5842 DE circRNAs in the MDA-MB-231 cells compared with the MCF-7 cells by cluster analysis (60). Compared with healthy controls, 1061 and 1084 exosomal circRNAs were upregulated, while 86 and 301 exosomal circRNAs were downregulated in patients with metastatic disease and patients with localized disease, respectively. They also obtained 432 pairs of circRNA/miRNA interactions to construct circRNA/pathway and circRNA/miRNA networks. However, these circRNA studies have some potential limitations, mainly lacking a larger sample confirmed by RT-PCR. In addition, the circRNA-miRNA interactions should be confirmed by using the luciferase reporter assay and the enriched pathways should also be evaluated by experimental methods, but not only by bioinformatic methods (60).

## Exosomal ncRNAs in Immunological Regulation

Exosomes are considered to be important mediators of cellular communication between the immune cell and cancerous cells (61). Tumor-immune cell interactions shape the immune cell phenotype, with miRNAs, lncRNAs, and circRNAs being crucial components of this crosstalk (**Figure 2B**). Jiang et al. proposed a novel interplay mode between cancer cells and immunocytes (62). BC tumor exosomal miR-9 and miR-181a activated the JAK/STAT signaling pathway by targeting SOCS3 and PIAS3 respectively, and thus consequentially promoting the development and immunosuppressive function of mice early-stage myeloid-derived suppressor cells (eMDSCs), resulted in T-cell immunity inhibition and tumor progress (62). Xing et al. demonstrated loss of XIST also augmented secretion of BC exosomal miRNA-503, which triggered M1-M2 polarization of microglia to upregulate immune-suppressive cytokines in microglia to suppress T-cell proliferation. Thus, the lncRNA XIST played a critical role in BCBM in a gender-specific manner by influencing both tumor cells and the TME (54).

miR-27a-3p has been acknowledged as an oncogenic RNA in various cancers and is one of the highly enriched miRNAs in BC-derived exosomes. Under endoplasmic reticulum stress, BC cells could produce exosomes containing a high level of miR-27a-3p, which up-regulated PD-L1 in macrophages and thereby promoted immune evasion of BC cells by activating the PTEN/AKT/PI3K axis (63). Jang et al. showed that exosomal miR-16 from 4T1 cells treated with epigallocatechin gallate (EGCG), could be transferred to tumor-associated macrophages (TAMs) *via* exosome, ultimately leading to reduced tumor growth by inhibiting TAMs infiltration and M2 polarization (64). Exosomes secreted by macrophages shuttle invasion-potentiating miRNAs into BC cells. In the BC cell and macrophage co-culture system, Yang et al. concluded that macrophages exosomal miR-223 promoted the invasion of BC cells *via* the Mef2c-β-catenin pathway (65). These results have also emphasized the importance of the exosome shuttled between BC cells and TAMs.

Additionally, Ni et al. revealed that BC could modify the CD73 expression on γδT cells in a non-contact manner. BC-derived exosomal lncRNA SNHG16 served as a ceRNA by harboring miR-16-5p to derepress SMAD5, resulted in the conversion of γδ1 T cells into the CD73+ immunosuppressive subtype for favoring BC progress (66). lncRNA BCRT1 was markedly up-regulated in BC tissues, which was associated with poor prognosis in BC patients. lncRNA BCRT1 competitively harbored with miR-1303 to inhibit the degradation of target gene PTBP3, resulted in promoted BC progression (67). Moreover, lncRNA BCRT1 could be transported to macrophages *via* BC cell exosomes, thereby promoting M2 polarization and enhancing tumor progression.

## Exosomal ncRNAs as Promising Diagnostic Biomarkers

Early detection, therapy, and metastasis monitoring are of great importance to a favorable prognosis. Conventional diagnostic programs, such as breast X-ray mammography, ultrasound imaging, and positioning biopsy, usually cause radioactive or invasive damage to patients with limited accuracy. As a non-invasive method, liquid biopsy is convenient for repeated sampling in clinical cancer prognostic, metastatic assessment, and recurrence monitoring. Aberrant expression of ncRNAs profiles in exosomes released into circulating systems especially in plasma and serum are promising candidate biomarkers for BC liquid biopsy.

Lots of studies showed that there were differential expressions of exosomal miRNAs in BC patients and healthy individuals. As miR-155 was up-regulated in the tumor cells of BC patients, particularly the patients with early-stage and TNBCs, the plasma miR-155 could serve as a non-invasive biomarker for non-invasive detection of early-stage BC (68). Given that approximately 20 to 25% of women diagnosed with localized BC (LBC) were subjected to neoadjuvant therapy, it was significant to screen effective biomarkers of liquid biopsy for the diagnosis and prediction of treatment response in BC patients with neoadjuvant therapy. By detecting the circulating tumor cells (CTCs) and serum exosomal miRNAs isolated from blood samples before or after neoadjuvant therapy, a study found that higher levels of exosomal miRNA-21, miRNA-222, and miRNA-155 were significantly correlated with the presence of CTCs, indicating that exosomal miRNAs and CTCs could be a complementary strategy for improving diagnosis and prognosis of patients with neoadjuvant therapy (69). However, in another study, miR-145, miR-155, and miR-382 in exosomes were isolated from the serum of BC patients and healthy donors but were not in a preferential manner in BC patients for detection (12). Although these studies both detected miR-155 as biomarkers in BC by PCR, it seemed to be a potential limitation with certain variations of results, which might attribute to sample differences, exosome abundance, and separation efficiency.

The gene information exchange between cells by exosome-mediated transfer may produce different miRNA patterns. Panels or combinations of specific exosomal miRNAs, combined with other conventional clinical biomarkers or pathological examinations, might improve the BC diagnostic efficiency. Based on a case-control study, Hirschfeld et al. confirmed a panel of 4-variable miRNA signature (miR-424, miR-423, miR-660, and let7-i) as a highly specific combinatory biomarker tool for distinguishing BC patients from healthy controls, with 98.6% sensitivity and 100% specificity (70). Similarly, Li et al. used qRT-PCR to identify 4 plasma miRNAs and 4 serum miRNAs from the miR-106a-363 cluster for BC diagnoses, such as miR-106a-3p, miR-106a-5p, miR-20b-5p, and miR-92a-2-5p, indicating the biomarker potential of exosomal miRNAs (71). By comparing the serum levels of exosomal miRNAs of 50 BC patients and 12 healthy donors, Eichelser et al. also indicated that exosomal miR-101 and miR-373 were linked to differentiation of BC from benign breast diseases (BBDs) or healthy individuals, and the expression level of exosomal miR-373 was higher in TNBC and more aggressive breast carcinomas (70).

Serum exosomal miR-148a might be a promising biomarker for the diagnosis and prognosis prediction for BC. Li et al. reported that serum exosomal miR-148a level was significantly reduced in patients with BC and the decreased exosomal miR-148a was associated with an unfavorable prognosis of BC (72). Li et al. profiled that exosomal miR-122-5p expression was distinctly up-regulated in BC plasma-derived exosomes compared with the adjacent normal tissue samples (73). Exosomal miR-122-5p might be useful as a supplement for the traditional BC diagnostic strategies. Plasma exosome-encapsulated miR-223-3p level was significantly associated with the malignancy of BC, showing the potential function for the early detection of invasive BC (22). Based on the nucleic acid-functionalized Au nanoflare probe, Zhai developed an in the situ detection method of miR-1246 level in human plasma exosomes (74). By using the exosomal miR-1246 as a marker, they successfully differentiated 46 BC patients from 28 healthy controls with 100% sensitivity and 92.9% specificity at the best cutoff (74). Lee et al. also constructed a method of *in situ* simultaneous detections of exosomal miR-21, miR-27a, and miR-375 by competitive strand displacement (75). This method of clinical serum samples detection could effectively distinguish BC patients from healthy donors. The exosomal miRNA profiles with high levels of cancer-associated sensitivity and specificity are hopeful indicators for prediction and early detection of BC, ultimately reflecting disease information of development, tumor burden, malignant progression towards metastatic recurrence, and drug resistance.

For lncRNA detection, Tang et al. showed that BC patients expressed higher serum exosomal HOTAIR than that in healthy individuals (76). Interestingly, the serum exosomal HOTAIR levels were also markedly decreased 3 months after surgery compared with the levels before surgery. In addition, high expression of exosomal HOTAIR caused a worse disease-free survival and overall survival (76). Zhong et al. detected the levels of lncRNA H19 in serum-derived exosomes by using quantitative RT-PCR (77). This work revealed that exosomal H19 levels were up-regulated in BC patients compared to that in patients with BBDs and healthy controls and that the median serum exosomal H19 levels were significantly decreased in post-operative than that in the pre-operative patients. Moreover, exosomal H19 expression levels were related to lymph node metastasis, distant metastasis, and TNM stages, all indicating the H19 potential as a non-invasive biomarker for BC diagnosis (77).

In conclusion, the exosomal ncRNAs for BC detection are mainly in tumor lesions and blood. Notably, most reports about detection are associated with miRNA, but the lncRNA and circRNAs are rarely studied. Compared with the other biomarkers, exosomal ncRNAs cargoes can avoid degradation by external proteases and other enzymes to keep the quantity and activation. Thus, as exosomal ncRNAs are of high stability, low complexity, and less invasive acquisition, the quantification and characterization of these ncRNAs can serve as a supplementary tool of the non-invasive detection for BC, ranging from early detection, recurrence prediction, metastasis assessment, and therapeutic outcomes.

## Drug Resistance

Currently, chemotherapeutic treatments based on hormones, cytotoxic agents, and targeted antibodies are frequently effective in controlling tumor growth and progression. The BC patients who initially respond to these therapies can develop resistance at a later stage. Chemoresistance is the most common obstacle for BC (78). Treatment resistance is not entirely determined by the intrinsic characteristics of tumor cells, but also by the simultaneous actions of many local microenvironmental factors. Emerging evidence emphasized that tumor cells, especially with the phenotype of malignancy and drug resistance, would secrete exosomes containing specific miRNAs, lncRNAs, and circRNAs to non-drug-resistant recipients like cancer cells, immune cells, and even normal stromal cells. Conversely, these nonneoplastic cells could also transmit the exosomes to the cancer cells, which encourages the erosion of cancer cells (79). Therefore, the gene modifications and information exchanges by tumorous exosomal ncRNAs are critical to therapeutic resistance for BC. Meanwhile, it would be very valuable to identify specific exosomal miRNAs involved in resistance to chemotherapeutic agents to make an early prediction warning to chemotherapy possible.

### miRNAs in Drug Resistance

Drug-resistant BC cells may spread resistance capacity to adjacent cells partially due to shuttled tumor-derived miRNAs-containing exosomes in TME, highlighting the importance of inhibiting the transfer of exosomal miRNA for BC drug resistance. According to the existing studies, adriamycin (ADR) is a common object for its effect on both survival improvement and inevitable drug resistance in BC. Adriamycin- and docetaxel-resistant BCa cells altered gene expression of sensitive cells and transmitted drug resistance by transferring specific miRNAs contained in exosomes (80, 81). Chen et al. analyzed the differential expression of exosomal miRNAs derived from ADR-resistant (A/exo) and parental BC cells (S/exo), showing that there were 52 novel miRNAs with high expression levels in A/exo involved in transcriptional misregulation in ADR-resistance (82). In another study, MCF-7/S transfected with miR-222 inhibitors lost resistance while miR-222 mimics could acquire ADR resistance, *via* exosome transmitting (83). Wei et al. also confirmed that the exosomal miR-221/222 enhanced tamoxifen resistance in recipient ER-positive BC cells, while anti-miR-221/222 blocked the propagation of tamoxifen resistance (84). It was consistent that exosomes from MCF-7/Adr and MCF-7/Doc mediated the resistance capacity spread, which was engendered by intercellular transfer of specific miRNA cargoes, represented by miR-222 (85). These results provided a new perspective for the boost of the effectiveness of tamoxifen on BC patients. Pan et al. reported that BC cell-derived exosomal miR-221-3p could promote the resistance of BC cells to ADR *via* regulating the PIK3R1-dependent PI3K/AKT signaling pathway both *in vitro* and *in vivo* (86). Likewise, Yu et al. found that miRNA-222 could be transferred from ADR-resistant BC cells to sensitive BC cells and bestowed them ADR-resistant ability by inhibiting the PTEN signaling pathway (83). Therefore, tumor-derived exosomes are effective in transmitting drug resistance and the delivery of exosomal miR-222 might be a mechanism. PIK3R1 was proven to be a target gene of miR-221-3p and was defined as a DE gene in invasive BC. Exosomal miR-221-3p mediated PIK3R1 downregulation promoted the resistance of BC cells to ADR by suppressing the PI3K/AKT signaling pathway both *in vitro* and *in vivo* (86). Li et al. confirmed that miR-770 was a prognostic biomarker in TNBC and was significantly decreased (87). Furthermore, miR-770 could antagonize the ADR-resistance and metastasis *via* targeting of STMN1 *via* regulation of apoptosis and EMT, and modified the TME *via* transportation to tumor-associated macrophage, which was also mediated by exosomes (87). Shen et al. verified that the chemotherapy-induced BC cells to secrete various exosome-contained extracellular vesicle miRNAs, such as miR-9-5p, miR-195-5p, and miR-203a-3p, by binding to ONECUT2 (88). Of particular, docetaxel treatment promoted the expression of miR-9-5p, miR-195-5p, and miR-203a-3p in circulating vesicles and improved the level of stemness-associated genes in mice xenograft mammary tumors, thus proposing the mechanism of interaction and self-adaptive survival of BC cells during cytotoxic therapy (88). In addition, the cisplatin-resistant MDA-MB-231 cell could secrete exosomes to enhance the resistance of recipient BC cells to cisplatin in an exosomal miR-423-5p-dependent manner (89). Through different mechanisms of exosomal miRNA, the resistant BC cell population may be crucial mediators to spread drug resistance features. Together, these findings provide encouraging insight into the exosomal-associated BC drug resistance.

In a clinical study, Del Re et al. addressed that in BC plasma-derived exosomes, the high baseline CDK4 mRNA levels were associated with the response to palbociclib plus hormonal therapy, while the increased expression of TK1 and CDK9 mRNA was associated with clinical resistance (90). It provided the first evidence that early increases in TK1, CDK4, and CDK9 expression of exosomes in HR+/HER2- mBC patients were significantly associated with poorer treatment response and disease progression. Zhong et al. collected preneoadjuvant chemotherapy biopsies and paired surgically-resected specimens and detected that 12 significantly up-regulated miRNAs may contribute to drug resistance of BC (91). O’Brien et al. screened miR-134 as the most substantially down-regulated miRNA that could be used as a biomarker for TNBC and as a potential therapeutic option (92). The miR-134-enriched exosome delivery resulted in HSP90 by targeting STAT5B, cellular migration and invasion, and enhanced sensitivity to anti-HSP90 drugs. Afterward, Chen et al. intended to analyze the miRNA signatures bioinformatically in exosomes from ADR-resistant and parental BC cells and identified the hub genes for up-expressed and down-expressed exosomal miRNAs such as CCND1 and PTEN (93). This bioinformatics study provided a comprehensive view of the function of dysregulated exosomal miRNAs, which might benefit overcoming ADR resistance in BC therapy.

Besides, CSCs are partially contributed to resistance against cancer therapy. Santos et al. found that miR-155 could be isolated from the exosomes of CSCs and resistant cells, followed by exosome transfer of resistant cells to the recipient sensitive cells (94). The result also established the significance of exosomal miR-155 in BC chemoresistance, with implications for targeting miR-155 signaling as a possible therapeutic strategy. Stromal communication by exosomes also orchestrated intricate crosstalk with BC cells to regulate therapy resistance. In accordance with the opinions, Boelens et al. successfully defined an exosome-activated antiviral pathway and cooperated with NOTCH3 to regulate the stroma-mediated expansion of treatment-resistant cells (95).

In summary, the current study of exosomal miRNAs may offer a new mechanism for the understanding of chemoresistance. Identification of BC-specific exosomal miRNAs and their underlying mechanisms will help determine sensitivity to therapeutic drugs and establish an appropriate therapeutic strategy in future BC treatment.

### lncRNAs in Drug Resistance

The trastuzumab dramatically improves the clinical prognosis of HER2-positive BC patients, but more and more patients finally become trastuzumab-resistant and experienced undesired progression. As lncRNA expression is critical for the treatment of HER2-positive BC, the trastuzumab-associated resistance may be partially due to the dysregulation of lncRNAs. For instance, Shi et al. identified that lncRNA-ATB up-regulated remarkably in trastuzumab-resistant SKBR-3 cells and the tissues of trastuzumab-resistant BC patients (96). As a mediator of TGF-β signaling, lncRNA-ATB could competitively bind to miRNA-200c and up-regulate the expression of ZEB1 and ZNF-217, resulted in trastuzumab resistance. In another study, the expression level of exosomal lncRNA-SNHG14 was up-regulated in the serum of patients who exhibited resistance to trastuzumab (97). The exosomal lncRNA-SNHG14 promoted the effect of trastuzumab *via* targeting the apoptosis regulator Bcl-2/Bax. In the *in vitro* assay, Chen et al. suggested that exosomal lncRNA AGAP2-AS1 could reduce the trastuzumab-induced cell death in sensitive cells (98). Moreover, AFAP1-AS1 was screened out to be up-regulated in trastuzumab-resistant cells compared to sensitive cells. For mechanism, exosomal AFAP1-AS1 could induce trastuzumab resistance by associating with AUF1 and promoting ERBB2 translation (99). Therefore, the AFAP1-AS1 level may be useful for predicting trastuzumab resistance. Wang et al. demonstrated that the intercellular transmission of H19 by exosomes conferred ADR resistance, while downregulation of H19 could sensitize ADR-resistant MCF-7 and MDA-MB-231 cells to ADR (100). It suggested that exosomal lncRNA H19 might be applied as a therapeutic target to overcome chemoresistance in BC patients. Xu et al. elucidated that tamoxifen-resistant BC cells LCC2 secreted lncRNA UCA1-overexpressed exosomes, which could cause resistance to tamoxifen treatment of the MCF-7 cells and decrease apoptosis through reduction of cleaved caspase-3 expression (101). Thus, the exosome-mediated transfer of lncRNA UCA1 led to increased tamoxifen resistance in BC cells. In addition, the high expression of exosomal HOTAIR in BC serum might be correlated with poor neoadjuvant chemotherapy and response to tamoxifen therapy (76).

### circRNAs in Drug Resistance

It is well-established that many vital signaling pathways are regulated by circRNAs in BC, specifically in chemoresistant cell lines and clinical samples. For instance, Yang et al. reported that circ-ABCB10 could contribute to paclitaxel resistance of BC cells, *via* up-regulating DUSP7 by capturing let-7a-5p (102). circBMPR2 might act as the sponge for miR-553 to relieve the inhibition of miR-553 on ubiquitin-specific protease 4 (USP4), thereby restraining the progression of BC and the resistance to tamoxifen (103). Hu et al. demonstrated that circ_UBE2D2 was upregulated in BC tamoxifen-resistant tissues and cell lines, and circ_UBE2D2 deletion mitigated tamoxifen resistance of BC cells (104). Furthermore, circ_UBE2D2 was significant enrichment in resistant cell-derived exosomes and shuttled into parental cells, resulted in enhanced tamoxifen resistance. More importantly, xenograft analysis displayed that exosomal circ_UBE2D2 also enhanced tamoxifen resistance *in vivo*. By targeting miR-200a-3p, tumorous exosome-mediated transfer of circ_UBE2D2 could enhance tamoxifen resistance of BC cells by regulating cell viability, metastasis, and the level of ERa *in vivo* and *in vitro*. However, up to date, the chemoresistance research on tumor-derived exosomal circRNA is rather little.

## Conclusions and Perspectives

Functionally, as vital mediators of cell-cell communication, ncRNAs-containing exosomes are pluripotent carriers with intriguing and elaborate roles in BC progression. After being released by parental cell exosomes, ncRNAs shuttle and transfer to neighboring or distant cells for reprogramming TME. In this review, it is highlighted that exosomal miRNAs, lncRNAs, and circRNAs participate in signaling pathways implicated in proliferation, apoptosis, invasiveness, EMT as well as in angiogenesis of recipients, thereby facilitating BC oncogenesis, proliferation, metastasis, and drug resistance (**Table 1**). Additionally, based on specific de-regulated expression verified by RNA sequencing, bioinformatic analyses, and PCR experiments, these tumor-derived exosomal ncRNAs that existed in blood samples are verified to be excellent candidates for improving early diagnosis, personalized prognosis, and therapeutic monitoring.

**Table 1:** The expression pattern, mechanism and clinical value of tumor-derived exosomal ncRNAs in breast cancer.

ncRNAs	Sample source	Expression	Mechanism	Clinical value	Ref.
miR-27a/b	Plasma	Upregulation	N/A	Potentially diagnostic and therapeutic markers for BC	(20)
miR-335		Upregulation			
miR-365		Upregulation			
miR-376c		Upregulation			
miR-382		Upregulation			
miR-422a		Deregulation			
miR-433		Upregulation			
miR-628		Upregulation			
miR-16	Plasma	Upregulation	N/A	Associated with estrogen and progesterone receptor status;	(21)
miR-30b	Plasma	Deregulation	N/A	Detection of BC recurrence;	
miR-93	Plasma	Upregulation	N/A	Detection of DCIS;	
miR−223−3p	Plasma BC tissues	Upregulation	N/A	A biomarker for detecting IDC initially diagnosed with DCIS by biopsy	(22)
miR-130a miR-425	BC cells	Upregulation	miR-130a and miR-425 may promote the proliferation of BC cells through TOR, ErbB, MAPK and TGF-Akt pathways.	N/A	(23)
miR-455-5p miR-1255a	BC cells	Upregulation	miR-455-5p may exert tumor promoting roles by inhibiting the expression of CDKN1B and influencing cell cycle and miR-1255a may be oncogenic by down-regulating SMAD4 and affecting TGF-β signaling pathway, which resulted in poor prognosis.	Prediction of poor prognosis	(24)
miR-31	BC cells	Upregulation	miR‐31 modulated the growth of MCF‐7 cells by targeting the HDAC2.	N/A	(25)
miR-9	BC cells	Upregulation	N/A	A therapeutic target	(27)
miR-3613-3p	CAFs BC tissues	Upregulation	The downregulation of miR-3613-3p expression could inhibit BC cell proliferation, ROS production and metastasis by targeting SOCS2.	A nonspecific diagnostic biomarker for BC and a potential biomarker for prognosis prediction of BC	(28)
miR-105	BC cells	Upregulation	miR-105 mediated metabolic reprogramming of CAFs promoted continuous tumor growth by regulating the Shared metabolic environment.	N/A	(29)
miR-221/222	CAFs	Upregulation	miR-221/222 was directly involved in ER inhibition and may be involved in MAPK-induced ER inhibition of BC cells.	Associate with aggressive breast cancer	(30)
miR-128	BC cells	Upregulation	miR-128 in exosomes negatively regulated the level of Bax in MCF-7 recipient cells and inhibits cell proliferation.	N/A	(32)
miR-1246	BC cells	Upregulation	miR-1246 could promote invasion in normal HMLE cells partially targeting CCNG2 by binding to its 3’-UTR.	A serum biomarker for BC	(33)
miR-222	Plasma BC cells	Upregulation	miR-222 promoted migration and invasion of the BC cells by repressing PDLIM2 expression and consequently enhancing NF-κB.	Correlated with BC metastatic progression	(34)
miR-9 miR-155	BC cells	Upregulation	miR-9- and miR-155-containing exosomes of highly metastatic MDA-MB-231 cells could decrease PTEN and DUSP14 expression in non-metastatic MCF-7 cell.	N/A	(35)
miR-193b	BC cells BC tissues	Deregulation	miR-193b could regulate the proliferation, invasion and migration of breast cancer cells through RAB22A.	A novel target for BC therapy	(36)
miR-10b	BC cells	Upregulation	The uptake of miR-10b could inhibit the protein levels of target genes HOXD10 and KLF4 in the receptor cells, and induce the invasion ability of non-malignant HMLE cells.	Targeting exosomal mirnas might provide an alternative approach for BC intervention	(37)
miR-939	BC cells BC tissues	Upregulation	miR-939 increased tumor cell trans-endothelial migration and regulated CDH5 in endothelial cells.	Association with worse prognosis in tnbcs	(38)
miRNA-210	BC cells Mice	Upregulation	miR-210 was involved in the expression of vascular remodeling related genes, such as Ephrin A3 and PTP1B, to promote angiogenesis in recipient cells.	Promoting angiogenesis	(40)
miR-130a-3p	BC tissues BC cells Blood samples	Deregulation	Overexpression of miR-130a-3p inhibited cell proliferation, migration, and invasion potentially *via* downregulation of RAB5B.	Associated with lymph node metastasis and advanced TNM stage	(41)
miR-454	BC cells Mice	Upregulation	miR-454 disrupted the Wnt pathway by targeting PRRT2, thereby promoting the biological properties of cancer stem cells.	N/A	(43)
miRNA-126	BC cells Mice	Deregulation	miRNA-126 strongly inhibited the formation of lung metastasis by blocking the PTEN/PI3K/AKT signaling pathway.	N/A	(44)
miR-9 miR-155	BC cells	Upregulation	miR-9 and miR-155 could down-regulate the expression of tumor suppressor genes PTEN and DUSP14.	N/A	(45)
miR-20a-5p	BC tissues BC cells	Upregulation	miR-20a-5p promoted osteoclast formation and bone metastasis by targeting SRCIN.	A more potential target for breast cancer therapy	(46)
miR-1246	BC cells Mice	Upregulation	miR-1246 might orchestrate the angiogenic niche in the brain.	N/A	(47)
lncRNA CASC9	BC tissues BC cells	Upregulation	lncRNA CASC9 regulated checkpoint kinase 1 (CHK1) by competitively binding to the miR-195/497 cluster, thereby accelerated BC cell proliferation.	A potential diagnostic marker for BC	(49)
lncRNA HOTAIR	BC cells Plasma tissue	Upregulation	N/A	A novel prognostic marker with liquid biopsy and thera- peutic targets	(50)
lncRNA MALAT1	Serum BC tissues BC cells Mice	Upregulation	N/A	A promising thera- peutic target for BC	(51)
lncRNA SNHG3	CAFs	Upregulation	The down-regulation of SNHG3 inhibited glycolytic metabolism and cell proliferation by increasing miR-330-5p and decreasing the expression of PKM in BC cells.	A novel target for cancer therapy	(52)
lncRNAGS1-600G8.5	BC cells Mice	Upregulation	lncRNA GS1-600G8.5 could disrupt the blood-brain barrier and promote the brain metastases, perhaps by targeting tight junction proteins.	A promising therapeutic target for brain metastasis *in vivo*	(53)
lncRNA XIST	BC cells BC tissues Mice	Deregulation	Downregulation of lncRNA XIST activated epithelial-mesenchymal transition, MSN/c-Met, and release of exosomal miR-503, accelerated primary tumor growth as well as metastasis in the brain.	An effective target for treating brain metastasis	(54)
hsa-circRNA-0088088	Serum	Upregulation	N/A	New insights into the prognosis and therapy of BC	(58)
has-circRNA-00000751	Serum	Upregulation			
hsa-circRNA-00005795	Serum	Deregulation			
miR-9 miR-181a	BC cells Blood samples BC tissues	Upregulation	miR-9 and miR-181a activated the JAK/STAT signaling pathway *via* targeting SOCS3 and PIAS3 respectively, resulted in T-cell immunity inhibition and tumor progress.	A potential therapeutic target for IL-6high breast cancer treatment	(62)
miR-27a-3p	BC cells BC tissues mice	Upregulation	miR-27a-3p up-regulated PD-L1 in macrophages and promoted immune evasion of BC cells by activating the PTEN/AKT/PI3K axis.	A novel therapeutic target for BC	(63)
miR-16	BC cells Mice	Deregulation	miR-16 reduced tumor growth by inhibiting TAMs infiltration and M2 polarization.	N/A	(64)
lncRNA SNHG16	BC cells BC tissues	Upregulation	lncRNA SNHG16 served as a ceRNA by sponging miR-16-5p to derepress SMAD5, resulted in the conversion of γδ1 T cells into the CD73+ immunosuppressive subtype for favoring BC progress.	Have potential for BC treatment	(66)
lncRNA BCRT1	BC tissues BC cells	Upregulation	lncRNA BCRT1 competitively harbored with miR-1303 to prevent the degradation of target gene PTBP3, resulted in promoted BC progression.	A potential biomarker and therapeutic target for BC.	(67)
miR-155	Plasma	Upregulation	N/A	A non-invasive biomarker for detection of early stage breast cancer	(68)
miRNA-21 miRNA-222 miRNA-155	Serum	Upregulation	N/A	A complementary clinical tool for improving breast cancer diagnosis and prognosis	(69)
miR-424 miR-423 miR-660 let7-i miR-373 miR-101	Serum Urine BC tissues	Upregulation	N/A	A promising non-invasive alternative	(70)
miR-106a-3p miR-106a-5p miR-20b-5p miR-92a-2-5p	Plasma Serum BC tissues	Upregulation	N/A	Promising novel biomarkers for the diagnosis of BC	(71)
miR-148a	Plasma BC tissues	Deregulation	N/A	A promising diagnostic and prognostic biomarker for BC	(72)
miR-122-5p	Plasma BC tissues	Upregulation	N/A	A promising biomarker for BC detection	(73)
lncRNA HOTAIR	Serum BC tissues BC cells	Upregulation	N/A	Correlated with poor neoadjuvant chemotherapy and response to tamoxifen therapy	(76)
lncRNA H19	Serum	Upregulation	N/A	A novel biomarker for the diagnosis of BC	(77)
miR-221/222	BC cells	Upregulation	N/A	A novel treatment strategy against tamoxifen resistance for BC	(84)
miR-221-3p	BC cells	Upregulation	miR-221-3p promoted the resistance of BC cells to ADR *via* the regulation of the PIK3R1-dependent PI3K/AKT signaling pathway.	A novel treatment strategy against tamoxifen resistance for BC	(86)
miR-770	BC cells BC tissues Mice	Deregulation	miR-770 promoted the chemo-sensitivity of TNBC to doxorubicin *via* induction of apoptosis.	A prognostic biomarker in triple negative BC	(87)
miR-9-5p miR-195-5p miR-203a-3p	BC cells BC tissues Mice	Upregulation	Exosomal miRNAs stimulated cancer stem cell-like features to induce chemical resistance in BC.	Inducing chemical resistance in BC	(88)
miR-423-5p	BC cells	Upregulation	N/A	A cisplatin resistance target	(89)
miR-134	BC cells BC tissues	Deregulation	The miR-134-enriched exosome delivery resulted in HSP90 by targeting STAT5B, cellular migration and invasion, and enhanced sensitivity to anti-HSP90 drugs.	A biomarker for TNBC and as a potential therapeutic option	(92)
miR-155	BC cells	Upregulation	N/A	A possible therapeutic strategy	(94)
lncRNA-SNHG14	Serum BC cells BC tissues	Upregulation	The exosomal lncRNA-SNHG14 promoted the effect of trastuzumab by targeting the apoptosis regulator Bcl-2/Bax.	A promising therapeutic target for patients with HER2+ BC	(97)
lncRNA AGAP2-AS1	BC cells	Upregulation	N/A	A therapeutic target	(98)
lncRNA AFAP1-AS1	BC cells BC tissues	Upregulation	Exosomal AFAP1-AS1 could induce trastuzumab resistance through associating with AUF1 and promoting ERBB2 translation.	Prediction of trastuzumab resistance and breast cancer treatment	(99)
lncRNA H19	BC cells Serum	Upregulation	N/A	A molecular target to reduce DOX resistance	(100)
lncRNA UCA1	BC cells	Upregulation	lncRNA UCA1-overexpressed exosomes, which could cause resistance to tamoxifen treatment of the MCF-7 cells and decrease apoptosis through reduction of cleaved caspase-3 expression.	N/A	(101)
circ_UBE2D2	BC cells Mice	Upregulation	By targeting miR-200a-3p, tumorous exosome-mediated transfer of circ_UBE2D2 could enhance tamoxifen resistance of BC cells.	Providing new insights into the boost of the effectiveness of tamoxifen on BC	(104)

ncRNAs, non-coding RNAs; N/A, not applicable; BC, breast cancer; DCIS, ductal carcinoma in situ; CAFs, cancer-associated fibroblasts; ROS, reactive oxygen species; ER, estrogen receptor; TNBC, Triple-negative breast cancer; TNM, tumor-node-metastasis; CHK1, checkpoint kinase 1; ADR, adriamycin.

Up to date, there are still some limitations and challenges in this subject. Firstly, the complex role of exosomal ncRNAs in BC progress remains to be further deeply explored. As TME is a complex entity, different types of cells secrete differential types of exosomal ncRNAs information, it would be an interesting angle that the specific factor is secreted by which cell type to exert the maximum or dominant impact in BC. Given the high level of genetic heterogeneity of BC tumors, understanding the pathogenesis of the key exosomal ncRNAs is meaningful to the development of new therapeutic strategies. Secondly, as the overwhelming priorities of BC-caused deaths result from metastasis, it is of great value to excavate new ncRNAs or exosomal ncRNAs for combating metastasis. It has been proved that BC-derived exosomes are involved in the key steps of primary tumor metastasis and spread, from the oncogenic reprogramming of malignant cells to the formation of the pre-metastasis niche. These effects are achieved by mediating crosstalk between cells and then modifying the local and remote microenvironment in an autocrine and paracrine manner. Identifying the key exosomal ncRNAs in the metastasis process will benefit the precision medicine-based strategies for diagnosis and therapy of BC metastasis (105). Thirdly, studies have emphasized that ncRNAs present an obvious asymmetric distribution in parent cells and their exosomes (106). It means that these regulatory RNAs are selectively encapsulated into exosomes during biogenesis. Coincidentally, Ni et al. suggested a selective and wavelike packaging of miR-16 into exosomes in the different BC subtypes possibly during tumor development and progression (21). Therefore, the DE ncRNAs in exosomes might be of the relatively slight or large difference in expression levels over their parental cells, but still reflecting the different pathological status of BC. However, the mechanism of selective packaging ncRNAs into exosomes needs to be elucidated more clearly. Fourthly, although numerous studies have addressed the characteristics of circRNAs in BC, there are still few reports regarding exosomal circRNAs. Most available circRNA biomarkers are currently not sensitive or specific enough to be applied clinically. The abundance, biogenesis and biological functions, and possible sorting mechanisms and potential roles of exosomal circRNAs in promoting or inhibiting cancer are worth further explorations.

It is also very important for utilizing tumor-derived exosomal ncRNAs as newly-developing diagnostic and therapeutic targets. The difficult part is that it is challenging to analyze candidate exosomal biomarkers in a routine clinical setting, mainly due to the lack of standardization for rapid assays, sources of variability, and methodological issues. Most blood-based biomarker studies are retrospective case-control studies, with small sample sizes, and different sample handling and storage methods due to lack of standardization and large-scale testing, lack of method validation, and non-exosomal contaminants like proteins existence (78). In addition, as sensitivity and specificity are two major standards for an accurate diagnostic tool, the optimal plasma/serum miRNA cut-off levels should be confirmed. In order to systematically prove the clinical utility as diagnostic, prognostic, and predictive biomarkers, it is necessary to quantify miRNAs in large-scale prospective multicenter studies and independent patient cohorts of different tumor stages.

Of particular, the ncRNAs, acting as regulators in shaping cellular activity, are believed to boost the clinical translations. Nevertheless, the promising strategies based on exosomal ncRNA in the field of personalized oncology are still in their infancy. Considering that exosomes are bioavailable, well-tolerable, targetable, and membrane-permeable, they are ideal candidates for delivering miRNAs, proteins, drugs, and other molecules to tumors. The suitability of exosomes as delivery vehicles for BC treatment has been investigated in recent years. For instance, Gong et al. yielded a novel exosome loaded with a chemotherapeutic adriamycin and cholesterol-modified miRNA, which exhibited effective anti-tumor function in MDA-MB-231 cell-represented cells and xenograft (107). Another treatment strategy may be to block the secretion of tumor cell-derived exosomes or to deliver therapeutic ncRNAs to the primary tumor through exosomes. Zhao et al. identified exosomes with lung targeting ability that derived from autologous BC cells accordingly constructed biomimetic nanoparticles CBSA/siS100A4@Exosome. These particles significantly inhibited the growth of malignant BC cells, showing a promising strategy to suppress postoperative BC metastasis (108). Ohno et al. also verified that exosomes were excellent candidates to therapeutically target EGFR-expressing cancerous tissues by efficiently delivering nucleic acid drugs, such as let-7a miRNA (109). Limoni et al. considered that exosomes were the best options for gene targeting and they established modified exosomes to target HER2-positive BC cells by loading siRNA (110). Up to the present, almost all the existing studies are still in the preliminary preclinical stage, including bioinformatics, sample sequencing, and testing, cell and animal experiments. That means that there are no clinically approved exosome-based therapeutics. The continuous exploration in fundamental researches and further clinical trials are urgent for the era of precision oncology in BC patients.

**Figure 3:**
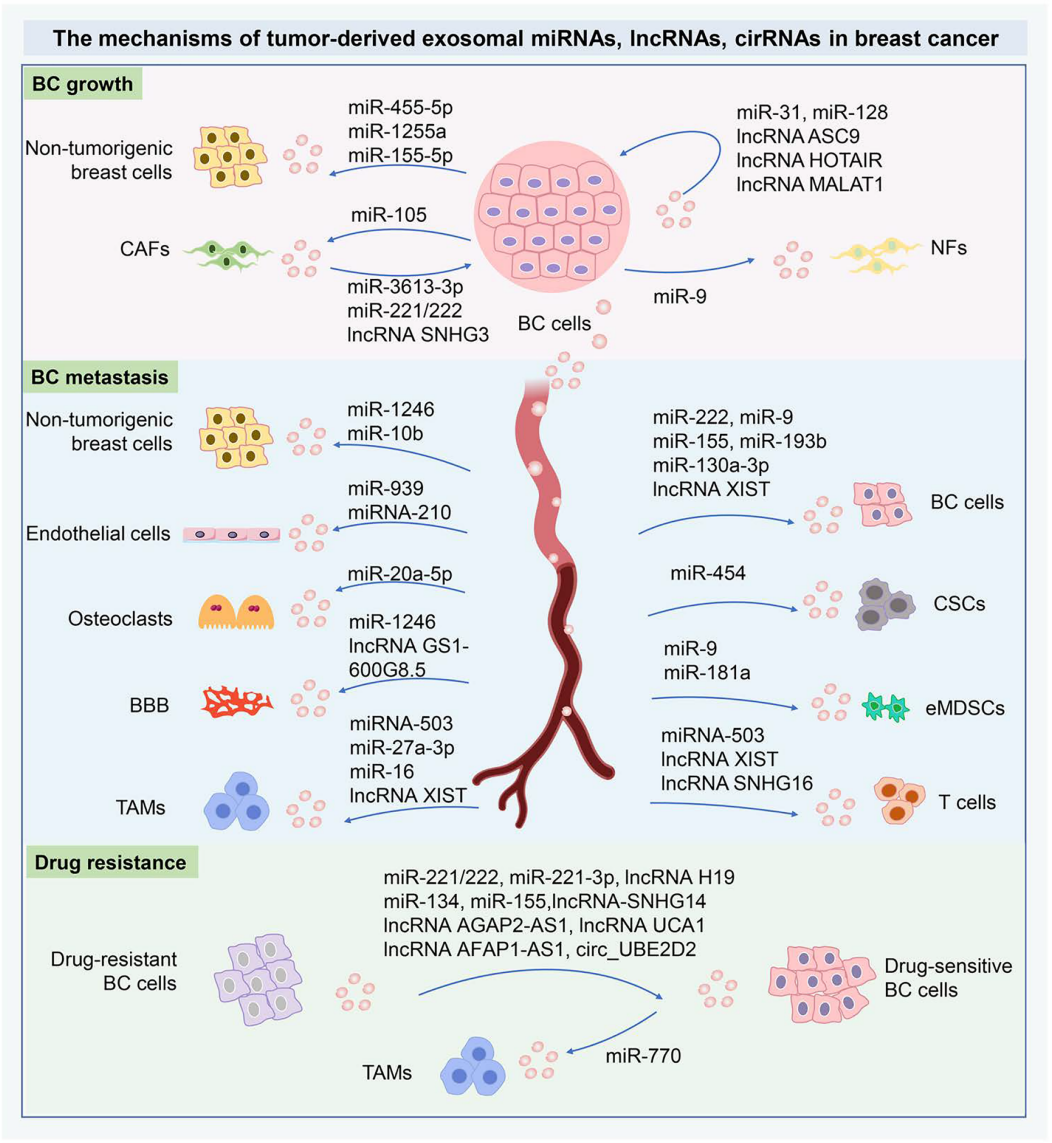
The mechanisms of tumor-derived exosomal miRNAs, lncRNAs, cirRNAs in BC. The tumor-derived exosomal miRNAs, lncRNAs, cirRNAs can shuttle cellular signals from the donor cells to adjacent recipients and then are internalized *via* exosome-mediated transmission. The tumor-derived exosomal ncRNAs are crucial orchestrators in the intercellular communication within the primary TME and metastasizing niche. The tumor-derived exosomal ncRNAs are essential to modify the cancer cell phenotypes locally or systemically through the exosome-dependent exchange of genetic information. In drug resistance, the characteristics of drug resistance can transfer from drug-resistant cells to drug-sensitive cells, therefore exosomal ncRNAs are critical to therapeutic resistance for BC. These important ncRNAs have been implicated as novel mediators of intercellular communication and as potential biomarkers in BC diagnosis and therapies. miRNAs, MicroRNA; lncRNA, Long-noncoding RNA; circRNAs, circular RNAs; BC, Breast cancer; CAFs, Cancer-associated fibroblasts; NFs, Normal fibroblasts; CSCs, Cancer stem cells; BBB, Blood-brain barrier; TAMs, Tumor-associated macrophages; eMDSCs, Early-stage myeloid-derived suppressor cells.

In conclusion, we offer the latest elucidation on the roles and mechanisms of tumor-derived exosomal ncRNAs in tumor growth, metastasis, detection, and drug resistance in BC (**Figure 3**). Further studies focusing on the tumor-derived exosomal ncRNAs will not only undoubtedly deepen the comprehensive understanding of their behavior, but also contribute to better clinical outcomes for BC-bearing individuals.

## Author Contributions

YY, MW, and HZ performed a literature search and wrote the manuscript. YW, QZ, and PD conceived the project and revised the manuscript. WH, CZ, MX, and WL edited the manuscript. All authors contributed to the article and approved the submitted version.

## Funding

This work was supported by China Guanghua Science and Technology Foundation (grant number 2019JZXM001) and Wuhan Science and Technology Bureau (grant 2020020601012241).

## Conflict of Interest

The authors declare that the research was conducted in the absence of any commercial or financial relationships that could be construed as a potential conflict of interest.

## Publisher’s Note

All claims expressed in this article are solely those of the authors and do not necessarily represent those of their affiliated organizations, or those of the publisher, the editors and the reviewers. Any product that may be evaluated in this article, or claim that may be made by its manufacturer, is not guaranteed or endorsed by the publisher.

## Abbreviations

BC, breast cancer; HER2, human epidermal growth factor receptor 2; EVs, extracellular vesicles; ncRNAs, non-coding RNAs; miRNAs, microRNA; lncRNA, long-noncoding RNA; circRNAs, circular RNAs; piRNAs, piwi-interacting RNAs; mRNA, messenger RNA; 3’-UTR, 3’-untranslated region; ECM, extracellular matrix; TNBC, Triple-negative breast cancer; DCIS, ductal carcinoma in situ; IDC, invasive ductal carcinoma; GEO, Gene Expression Omnibus; HDAC2, histone deacetylase 2; CDK2, cyclin-dependent kinases 2; DE, differentially expressed; TME, tumor microenvironment; NFs, normal fibroblasts; CAFs, cancer-associated fibroblasts; ROS, reactive oxygen species; ER, estrogen receptor; LV, lymphatic vessels; LECs, lymphatic endothelial cells; TNM, tumor-node-metastasis; BCSCs, breast cancer stem cells; CSCs, cancer stem cells; CAAs, cancer-associated adipocytes; BCF, BC specific frequencies; ceRNAs, competing endogenous RNAs; CHK1, checkpoint kinase 1; RTK, receptor tyrosine kinase; BBB, blood-brain barrier; EMT, epithelial-mesenchymal transition; BCBM, breast cancer brain metastasis; RT-PCR, reverse transcription-polymerase chain reaction; NGS, next-generation sequencing; eMDSCs, early-stage myeloid-derived suppressor cells; EGCG, epigallocatechin gallate; LBC, localized BC; CTCs, circulating tumor cells; BBD, benign breast disease; ADR, adriamycin; USP4, ubiquitin-specific protease 4.
